# Recent technological advancements in membrane distillation and solar stills: preheating techniques, heat storage materials, and nanomaterials — a detailed review

**DOI:** 10.1007/s11356-022-19625-w

**Published:** 2022-03-16

**Authors:** Mohamed Abdelgaied, Mohamed Fathi Seleem, Mohamed Mahgoub Bassuoni

**Affiliations:** Mechanical Power Engineering Department, Faculty of Engineering, Tanta University, Tanta, Egypt

**Keywords:** Membrane distillation, Solar stills, Preheating technology, Thermal storage mediums, Nanomaterials, Performance improvement

## Abstract

Freshwater and energy are critical components for the growth and progress of societies. The scarcity of freshwater and rapid population growth, especially in remote countries, has led to an urgent need to develop desalination technologies in order to raise its productivity and reduce its energy consumption rates. Membrane distillation is one of the effective methods characterized by its high productivity, but its disadvantage by higher electricity consumption. Also, solar stills are one of the sustainable and economical technologies, but the disadvantage by lower productivity. Accordingly, this manuscript dealt with a comprehensive review and detailed comparison of the most important modifications and innovations that were made to the design of the membrane distillation units, which aim to reduce electricity consumption rates, as well as the design of solar stills, which aims to maximize the productivity and efficiency. This was done by providing a detailed comparison of the most important three axes of modifications and innovations that were addressed by recent previous studies on the design of membrane distillation units and solar stills, and their statement as follows: preheating technology, use of the thermal storage materials, and nanomaterials technology. Finally, based on this review, the authors make some recommendations for future work in the field of solar and membrane desalination.

## Introduction

Water is the most valuable and vital natural resource of all species. Fortunately, natural water is the most abundant compound on Earth, covering about 71% of the Earth’s surface (USGS 2019). Despite this, natural freshwater resources around the world are very scarce (Mekonnen and Hoekstra 2016). This is because about 97% of the water on Earth is in the oceans with a salinity range of 3.5–5%. Only 2.5% of the world’s total water resources are fresh; and only 31.4% of them can be accessed to support life on Earth (Powers 2017). Freshwater resources are available either as groundwater in the ground or as surface water in rivers, lakes, etc. In recent years, the stress on the available freshwater resources has been exacerbated as a result of many factors, the most important of which are: population growth, climatic changes, urban expansion, and changing lifestyles and prosperity. About 2.1 billion people during the current time period lack access to safely managed drinking water (UN-Water 2018; Xiao et al. 2021; Altarawneh et al. 2020). It is expected that by 2030, about 700 million people will be displaced worldwide due to the scarcity of fresh water (Wei et al. 2021; Al-Otoom and Al-Khalaileh 2020; Sharon et al. 2020). To overcome the problem of fresh water scarcity, some strategies have been developed that aim to reduce the demand for fresh water by implementing conservation practices or adjusting prices. However, previous strategies to mitigate fresh water demand do little to provide optimal solutions in highly affected regions (Gude 2017; Pourafshar et al. 2020; Siddiqui and Dincer 2018). On the other hand, the use of desalination technologies has enabled us to meet the increasing demand for fresh water by making use of the abundant reserves of brackish water and/or seawater.

Among the available desalination techniques, the solar distillation system is an effective procedure that relies on solar energy to produce fresh water, as it is built using locally available materials and also enjoys preserving depleted energy sources by using renewable resources (solar energy), which is clean and environmentally friendly energy (Manchanda and Kumar 2018; Velmurugan and Srithar 2011). Membrane-based desalination also has some advantages such as modularity, compactness, and sometimes the use of solar energy, thus preserving depleted energy sources by using renewable resources (solar energy), which is clean and environmentally friendly energy compared to multi-effect distillation (MED), thermal vapor compression (TVC), mechanical vapor compression (MVC), multi-stage flash distillation (MSFD), electro dialysis (ED), reverse osmosis (RO), freezing, and humidification and dehumidification (HDH) (Velmurugan and Srithar 2011). Despite the advantages of solar stills, the productivity is lower compared to the traditional desalination system. Membrane distillation (MD) has a lot of advantages such as it is operating with low-grade thermal energy and low pressures and large contact area; however, it also has some disadvantages especially high-power consumption which made it essential to reduce the power consumption and increase the efficiency of MD to make it even more practical. As a result of the high energy consumption of MD, it was found that it urgently needs renewable energy sources, heat storage mediums, and new configurations in membrane modules to reduce the required energy and improve the efficiency of MD (Gonzáleza et al. 2017). Ding et al. (2005) analyzed a solar-powered membrane distillation system; results have shown that the plant capacity in June can reach about 300 kg/day with solar energy. Another effective way of water desalination in remote areas is using solar stills. Solar stills use the radiation from the sun to evaporate water in a basin and then the water vapor condensate and accumulate as pure water. Solar stills productivity is affected by different factors like wind speed, solar rays, ambient temperature, glass-water temperature difference, absorber area, water-free surface area, water inlet temperature, basin water depth, and glass angle. The solar intensity, wind velocity, and ambient temperature cannot be controlled as they are meteorological parameters, whereas the remaining parameters can be controlled to improve productivity (Sivakumar and Sundaram 2013). Therefore, a great effort has been made in recent years to develop solar desalination systems, membrane-based desalination systems, as well as research into sustainable and energy-saving methods for fresh water production.

The rates of total energy consumption of desalination units depend on the design of the plant, the type of desalination method, the salinity of the feed water, and the temperature of the feed water. Thermal methods of water desalination require thermal energy and electrical energy, which makes their energy consumption higher than membrane methods that require only electrical energy to desalinate water. Therefore, the rate of total energy consumption required to desalinate 1 m^3^ of fresh water varies according to the desalination method used, where the total energy consumption required for MED system ranges between 14.45 and 21.35 kWh/m^3^, for MFS system between 19.58 and 27.25 kWh/m^3^, for TVC system 16.3 kWh/m^3^, for MVC system between 7 and 12 kWh/m^3^, and for ED system it is between 0.7 and 5.5 kWh/m^3^ (Maleki et al. 2016; Okampo and Nwulu 2021), while the total energy consumption for the RO system ranges between 2 and 4 kWh to produce one cubic meter of fresh water (Maleki et al. 2016; Okampo and Nwulu 2021).

The present manuscript dealt with two very important axes. The first axis dealt with the comprehensive review and a detailed comparison of the most important modifications and innovations that have been made to the design of membrane distillation units, which aims to reduce energy consumption rates as the following: feed water preheating technology, use of the thermal storage materials, and nanomaterials technology. Also, the second axis dealt with a comprehensive review and detailed comparison of the most important modifications and innovations that were made to the design of solar stills, all of which aim to maximize productivity and efficiency as the following: pre-heating technology, use of the thermal storage materials, and nanomaterials technologies. Finally, based on this review, the authors make some recommendations for future work in the field of solar and membrane desalination.

## Membrane distillation

### Overview

During the last few years, membrane desalination showed a high capability to produce distilled water. Membrane distillation is a dual technology that combined the advantage of thermal distillation and membrane separation (Anvari et al. 2020). Membrane distillation (MD) is classified according to its operational method into; direct contact membrane distillation (DCMD), sweeping gas membrane distillation (SGMD), air gap membrane distillation (AGMD), and vacuum membrane distillation (VMD) (Anvari et al. 2020). The advantages of membrane distillation over other desalination processes are concentration polarization, high rejection of non-volatile compounds, limited fouling, low operating temperature, and low operating pressure (Anvari et al. 2020). Therefore, membrane distillation technology has gained great attention for processes of desalination and treatment of wastewater (Anvari et al. 2020).

### Heat transfer in MD process

**Fig. 1:**
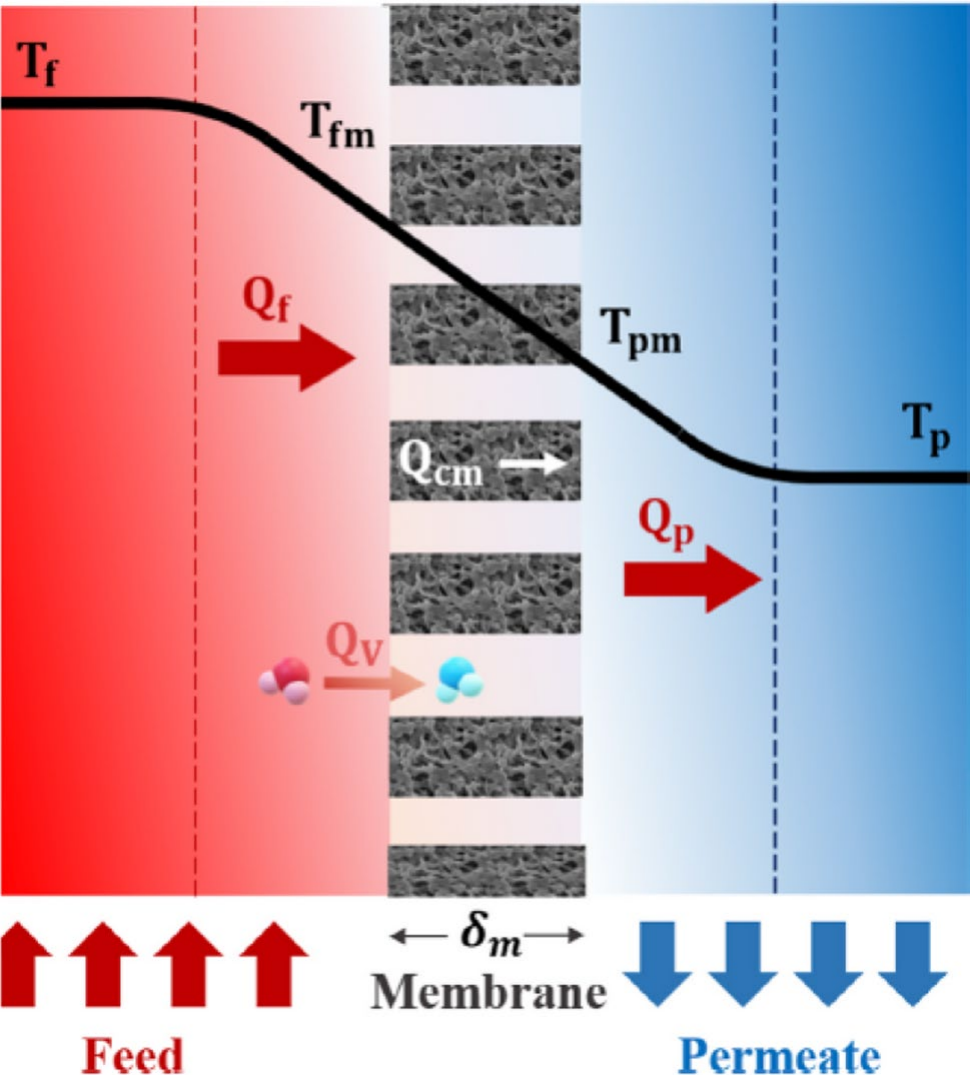
Heat transfer mechanisms in direct contact membrane distillation system

The performance of membrane distillation technology depends on the temperature gradient between the feed side and the distillate side, which results in the transfer of heat from the hot side of the membrane to the cold side, and thus the water phase change from a liquid to a vapor state on the surface of the feed side of the membrane. As shown in Fig. 1, the heat transfer through the membrane occurs in three regions: the feed layer Q_f_, the membrane Q_m_, and the permeate layer Q_p_, calculated using Eqs. (1)–(3).

1 $${\mathrm Q}_{\mathrm f}={\mathrm h}_{\mathrm f}\left({\mathrm T}_{\mathrm f}-{\mathrm T}_{\mathrm{fm}}\right)$$

2 $${\mathrm{Q}}_{\mathrm{m}}={\mathrm{Q}}_{\mathrm{cm}}+{\mathrm{Q}}_{\mathrm{v}}=\frac{{\mathrm{k}}_{\mathrm{m}}}{{\updelta }_{\mathrm{m}}}\left({\mathrm{T}}_{\mathrm{fm}}-{\mathrm{T}}_{\mathrm{pm}}\right)+\mathrm{J}\Delta {\mathrm{H}}_{\mathrm{v}}$$

3 $${\mathrm{Q}}_{\mathrm{p}}={\mathrm{h}}_{\mathrm{p}}\left({\mathrm{T}}_{\mathrm{pm}}-{\mathrm{T}}_{\mathrm{p}}\right)$$

where; h_f_ is the coefficient of heat transfer on feed side, h_p_ is a coefficient of heat transfer on permeate side, T_f_ is a feed temperature, T_fm_ is a membrane surface temperature in feed side, T_p_ is a permeate temperature, T_pm_ is a membrane surface temperature in permeate side, k_m_ is a membrane thermal conductivity, δ_m_ is the membrane thickness, J is the membrane flux, and ΔH_v_ is the evaporation enthalpy.

The overall heat transfer through the three regions Q is calculated as follows:

4 $$Q={Q}_{f}={Q}_{m}={Q}_{p}$$

5 $$\mathrm{H}\left({\mathrm{T}}_{\mathrm{f}}-{\mathrm{T}}_{\mathrm{p}}\right)={\mathrm{h}}_{\mathrm{f}} \left({\mathrm{T}}_{\mathrm{f}}-{\mathrm{T}}_{\mathrm{fm}}\right)=\frac{{\mathrm{k}}_{\mathrm{m}}}{{\updelta }_{\mathrm{m}}}\left({\mathrm{T}}_{\mathrm{fm}}-{\mathrm{T}}_{\mathrm{pm}}\right)+\mathrm{J}\Delta {\mathrm{H}}_{\mathrm{v}}={\mathrm{h}}_{\mathrm{p}}\left({\mathrm{T}}_{\mathrm{pm}}-{\mathrm{T}}_{\mathrm{p}}\right)$$

Coefficient of overall heat transfer H is calculated as follows:

6 $$\frac{1}{H}=\frac{1}{{h}_{f}}+\frac{1}{\frac{{\mathrm{k}}_{\mathrm{m}}}{{\updelta }_{\mathrm{m}}}+\frac{J\Delta {H}_{v}}{\left({T}_{fm}-{T}_{pm}\right)}}+\frac{1}{{h}_{p}}$$

The membrane surface temperatures on feed and permeate sides T_fm_ and T_pm_ are calculated as follows:

7 $${T}_{fm}={T}_{f}-\frac{\pi \frac{{\mathrm{k}}_{\mathrm{m}}}{{\updelta }_{\mathrm{m}}}\left({\mathrm{T}}_{\mathrm{fm}}-{\mathrm{T}}_{\mathrm{pm}}\right)+\mathrm{J}\Delta {\mathrm{H}}_{\mathrm{v}}}{{h}_{f}}$$

8 $${T}_{pm}={T}_{p}-\frac{\pi \frac{{\mathrm{k}}_{\mathrm{m}}}{{\updelta }_{\mathrm{m}}}\left({\mathrm{T}}_{\mathrm{fm}}-{\mathrm{T}}_{\mathrm{pm}}\right)+\mathrm{J}\Delta {\mathrm{H}}_{\mathrm{v}}}{{h}_{p}}$$

### Membrane distillation performance enhancement techniques

The most encountered problem in membrane desalination is that it requires high power consumption; a lot of research investigated the performance of membrane desalination with different techniques of improvement to reduce the power consumption and increase the productivity of the membrane distillation. Preheating the inlet water to the membrane is one of the most common methods. Preheating can be done by external heaters like electrical heaters, and it can take place by using solar energy to preheat the water inlet by using solar preheaters, which in turn can reduce the power consumption cost of the electric heater, and it is a renewable and cleaner source of energy (Shafieian and Khiadani 2019). Another very effective way to improve the membrane performance is using thermal storage mediums especially, with solar-driven membranes as the thermal storage medium works as a heat supply for preheating the feed flow when the solar irradiance decreases (Abdelgaied et al. 2020). New configurations of membranes with nanomaterials can also be used to improve membrane performance (Elango et al. 2015).

### Feed-water preheating technology

**Fig. 2:**
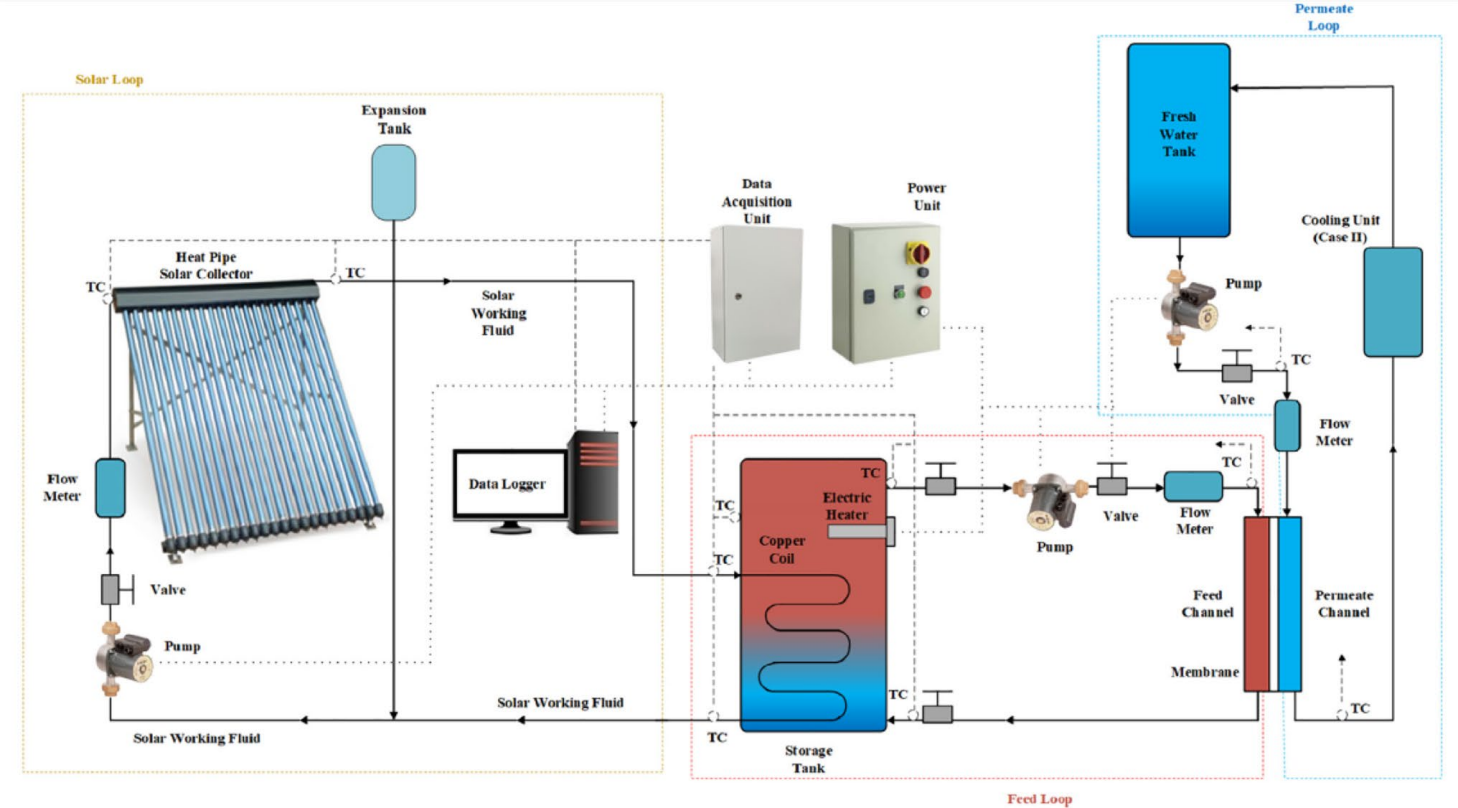
Layout of solar-driven membrane distillation (Shafieian and Khiadani 2019)

**Fig. 3:**
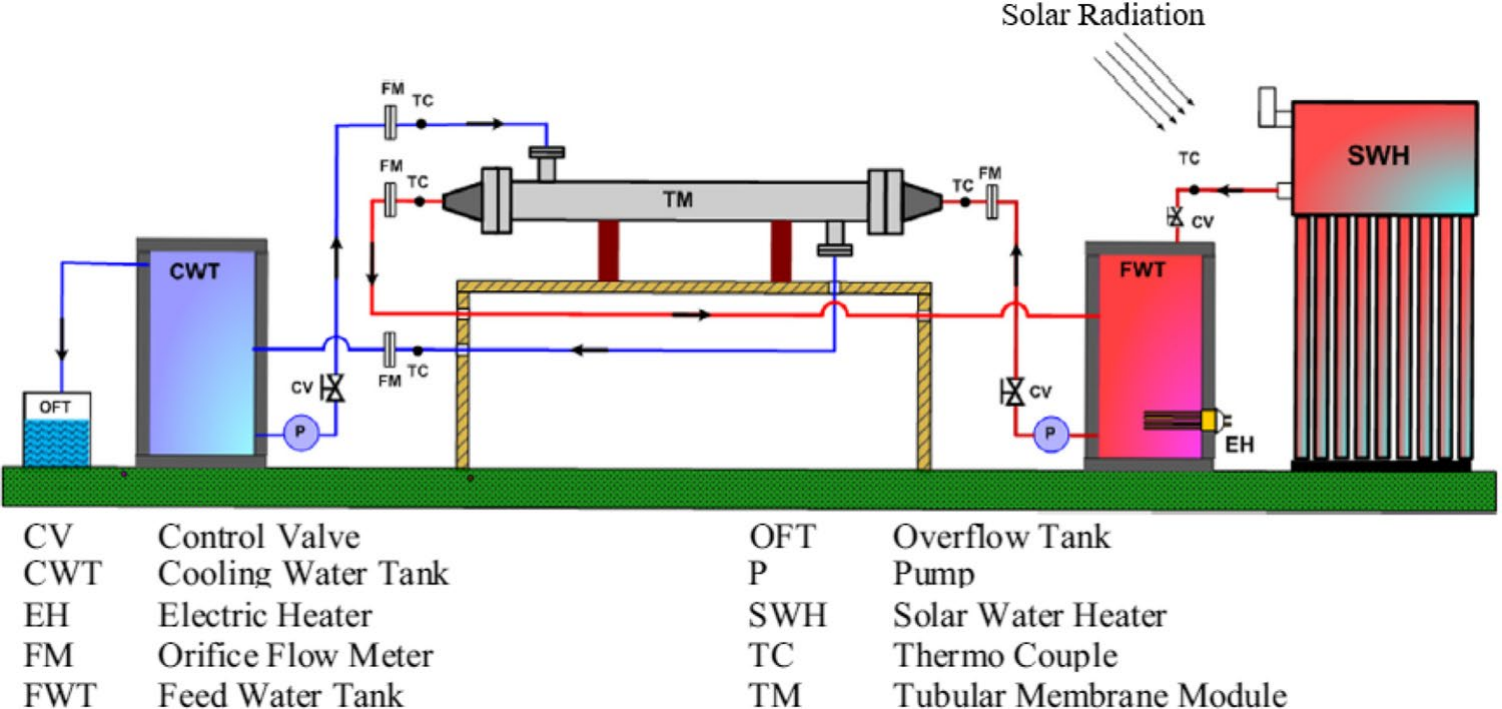
Schematic of direct contact membrane distillation assisted by solar energy (Elzahaby et al. 2016)

Different researches were done on preheating the feed water before its entering the membrane to increase the vapor pressure difference across the membrane sides which in turn increases the productivity. Shafieian and Khiadani (2019) studied experimentally and theoretically the behavior of the thermal-driven direct tubular contact membrane shown in Fig. 2 by using an electric heater to preheat the feed water before entering the membrane. They found that lower permeate temperature, as well as, higher feed water temperature results in higher freshwater production, and improves the freshwater production of the tubular DCMD unit. The system consists of three main loops including the solar heating loop, membrane feed loop, and membrane permeate loop. Three different operating cases were investigated (summer without cooling unit (Case I), summer with cooling unit (Case II), and winter without cooling unit (Case III)). They found that except for a few minutes in the morning, the solar collector was able to provide all required thermal energy to heat the feed water before entering the membrane unit. By adding permeate water cooling unit in the summer, the maximum production rate will be increased from 2.78 L/m^2^ h in Case I to 3.81 L/m^2^ h in Case II, as well as the overall efficiency of the system will be improved from 46.6% in Case I to 61.8% in Case II. Elzahaby et al. (2016) studied a direct contact membrane distillation system assisted by a cooling water tank and solar energy shown in Fig. 3. Effect of salt concentration, feed temperature, feed flow rate, the cooling temperature was investigated. They conducted that the water production rate increases with increasing the feed temperature; however, in this study, to avoid scale formation, the feed temperature is limited to 70 $\mathrm{^\circ{\rm C} }$. Recently reported studies examining the effect of feed water preheating systems on the performance of membrane distillation systems are summarized in Table 1.

**Table 1:** Recent studies on the performance of membrane distillation systems using feed water preheating systems

Refs	Nature	Improvement-techniques	Results	Remarks
Shafieian et al. (2019)	Experimental + Simulation	Feed preheating using electric heater	Permeate water increase by 52 g/m^2^ min for increasing feed temperature from 30 to 60 °C	It is more efficient to heat the feed water stream to improve water productivity than to use the same amount of energy to cool the permeate stream
Shafieian and Khiadani (2019)	Experimental	Feed preheating using evacuated tube solar water collector + Electrical heater	• Permeate water productivity reached 3.81 L/m^2^ h with cooling unit • Maximum thermal efficiency of the solar system reached 78% • Exergy efficiency varying between 4 and 5% • Overall system efficiency improved from 46.6 to 61.8% for using the cooling unit in the permeate flow loop • Solar working fluid temperature varying between 37 and 58 °C	Except 15 min in the morning, the heat pipe solar collector was able to operate the desalination system independently without any additional required thermal energy
Elminshawy et al. (2020)	Experimental + Simulation	Electric heater + V-trough solar concentrator PV panels with cooling + Buried water heat exchanger	For feed water of 80 $$\mathrm{^\circ{\rm C} }$$ and 144 kg/h, the permeate flux is 0.76 kg/$${\mathrm{m}}^{3}$$ h, Specific thermal energy consumption is 103 kWh/m^3^, GOR is 1.36, and produced water cost reached 22.48 $/m^3^	The hybrid system has the capacity to produce 19.58 m^3^ of freshwater per year at a cost of 22.48 $/m^3^ and to reduce CO_2_ emissions by 136.82 kg
Wang et al. (2019)	Experimental	Solar PV with thermal recovery integrated with MD	Pure water productivity reached 3.25 kg/m^2^.h for utilizing 5-stage MD integrated with PV	This device can transform an electric power plant from a water consumer to a pure water producer
Ding et al. (2005)	Experimental + Simulation	Feed preheating using evacuated tube solar water collector	About 23.5 l/h average water productivity for 0.8 l/min feed flow rate and feed temperature 70 °C	-
Elzahaby et al. (2016)	Experimental + Theoretical	Feed preheating using evacuated tube solar water collector + Electrical heater	• The daily productivity of pure water reaches 40.587 kg/day for 20 L/min feed flow rate and feed temperature 70 °C • Daily efficiency and Gain output ratio reached 60.06% and 0.624	-
Kabeel et al. (2017a)	Experimental	Feed preheating using evacuated glass tube solar water collector + Evaporative cooler	• Maximum productivity reached 33.55 L/day • System efficiency reached 49.01% and gain output ratio reached 0.49 • Feed temperature ranged 55–70 °C	Use of the cooling unit on permeate flow loop improved the system productivity almost 1.25
Soomro and Kimc (2018)	Theoretical	Solar power tower plant to produce the electricity and preheated the seawater before supplied to MD	• The maximum permeate flux 29.05 kg/$${\mathrm{m}}^{2}.$$ h was achieved at feed temperature 45 °C • The average freshwater produced up to 40,759 L/day • Estimated water cost 0.392 $/m^3^	• Increasing the feed temperature increased the permeate flux • The effect of the feed flow rate is not significant compared to permeate flow rate
Siefan et al. (2022)	Experimental	Feed preheating using flat plate solar collector + Solar-powered PV collectors	• Solar powered was a better option for membrane distillation in terms of an environmental footprint	-
Sandid et al. (2021)	Experimental and simulation	Feed preheating using flat plate and evacuated tube collectors + electric heater	• The specific thermal energy consumption ranged from 158.83 to 346.55 kWh/m^3^ • The hot feed inlet temperature ranged 50–65 °C • The maximum gain output ratio reaches 4.4 • Thermal efficiency reached 72% • Cost of fresh drinking water reached 14.73 $/m^3^	Using solar energy reduces carbon dioxide emissions by 7274.45 kg/year
Chang et al. (2022)	Practicality	Feed preheating using evacuated tube solar collector	Permeate flux reached 5.2 kg/m^2^ h at feed temperature 52 °C	This system is very effective for remote areas and especially for coastal fishery communities
Usman et al. (2021)	Economic feasibility	Feed preheating using thermal solar collector and waste heat recovery	• Increase the membrane permeability for using solar-thermal and waste heat • Reduced the rate of external power required to operate the system from 40 to 60% • Decreased water price from 6.80 $/m^3^ (the cost of operating the system with the electricity only) to only 1.6 $/m^3^	The contribution of solar heat and waste heat used in the operation of the process leads to a lower cost of water production as well as making the desalination system more competitive, sustainable and economically viable for small and remote applications
Gustafson et al. (2018)	Theoretical	Waste heat + Chiller	Permeate water flux reached 22.9 L/m^2^ h at feed inlet temperature 64 °C and distillate temperature of 30 °C	Membrane productivity depends strongly on waste heat source characteristics
Abdelkader et al. (2019)	Experimental	Electrical heater	Permeate flux reached 13 kg/m^2^ h at a water temperature difference of 30 °C	-

### Membrane with thermal storage mediums

**Fig. 4:**
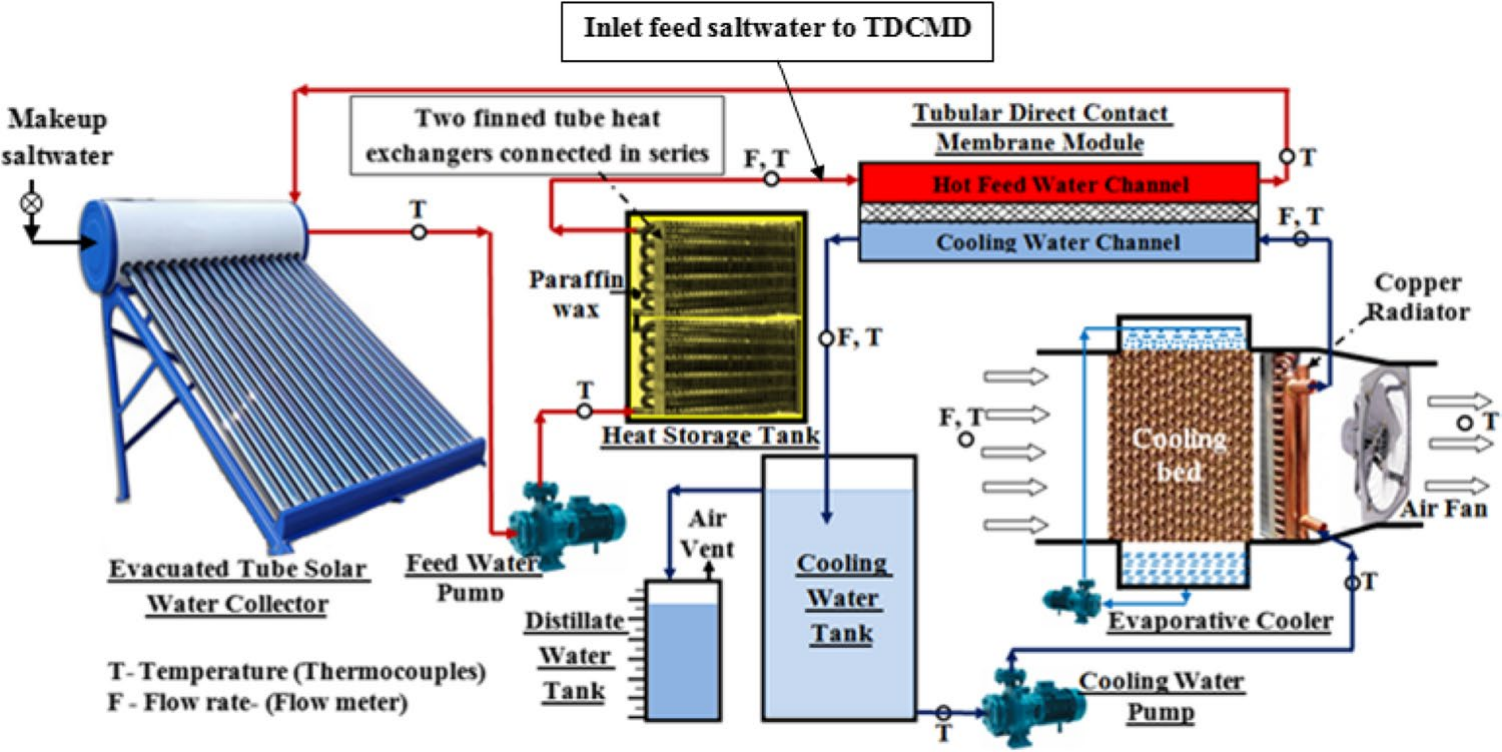
Test rig of solar-powered membrane distillation with energy storage mediums (Abdelgaied et al. 2020)

Thermal storage mediums are used in membrane desalination systems to store waste heat from any other process. It is often used with solar systems as it stores heat from the sun in daytime and use it as heat source for feed flow of membrane at night. Thermal storage mediums can be sensible like molten salt or latent like paraffin wax. Abdelgaied et al. (2020) built a test rig shown in Fig. 4 to improve a behavior of solar-assisted membrane distillation using the energy storage medium as paraffin wax. They conducted that the freshwater production rate is varying between 3.47 and 4.35 l/h at a feed flow rate of 16 l/min, respectively. Also, the gain output ratio reached 1.123 and 1.25 for 12 and 16 l/min feed flow rate, respectively. Recently reported studies examining the effect of thermal storage mediums on the performance of membrane distillation systems are summarized in Table 2.

**Table 2:** Recent studies on the performance of membrane distillation systems using heat storage materials

Ref	Nature	Improvement-techniques	Results
Abdelgaied et al. (2020)	Experimental	Evacuated tube solar collector + Paraffin wax as energy storage materials (Solid/liquid heat capacity 2.95/2.51 kJ/kg °C) + Evaporative cooling	• For utilizing the Paraffin wax as energy storage materials, the productivity will be improved by a rate varying between 33.11 and 43.18% compared to cases without storage materials • GOR for a solar TDCMD with EC reached 0.93. While adding the Paraffin wax increases the GOR to 1.25
Chafidz et al. (2014)	Experimental	Evacuated tube solar collector + Thermal storage tank contains hot water + heat pump driven by PV cell	15.39 L/h average productivity with feed temperature of 69 °C
Chang et al. (2012)	Theoretical	Flat plate solar collector + Thermal storage tank contains hot water	0.12 kg water productivity in 6 h
Gustafson et al. (2018)	Experimental + Theoretical	Waste heat + Chiller + Thermal storage tank contains hot water	The higher water flux occurs when the waste heat source is on because it stores a greater quantity of heat at a faster rate
Kim et al. (2021)	Theoretical	Feed preheating using flat plate and evacuated tube collectors + Thermal storage tank with phase change materials (Sodium alum (NaAl(SO_4_)_2_·12H_2_O) with heat capacity of 181 kJ/kg) with 28-stage vacuum membrane distillation	• Gain output ratio of 28-stage system reached 47% • System performance improved with increasing number of stage • The produced fresh water cost reached 0.97 $/m^3^

### *Nanomaterials in **membrane distillation*

Nanomaterials can be described as a material that is in one dimension is less than 100 nm. At this size, material properties may change on the chemical and physical sides. Nanomaterials are characterized by a large surface area, more strength, and stability. Carbon-based nanomaterials and nano-zeolite will be discussed as they showed a remarkable improvement in water desalination.

### Carbon-based nanomaterials

Carbon-based nanomaterials such as carbon nanotubes and graphene have been widely used for their important features like high surface area, high thermal conductivity, have a low thermal expansion coefficient, and high mechanical strength. These properties, especially the high thermal conductivity, tend to improve the performance of membrane productivity and salt rejection. Bhadra et al. (2013) utilized the carboxylated nanotubes which are more polar to improve the productivity of membrane distillation (MD), a sweep gas membrane with 1 L/min dry air was used and results showed a flux reaching 19.2 kg/${\mathrm{m}}^{2}$ with salt rejection 99%. Recently reported studies examining the effect of carbon-based nanomaterials on the performance of membrane distillation systems are summarized in Table 3.

**Table 3:** Recent studies on the performance of membrane distillation systems using carbon-based nanomaterials

Reference	Material used	Operating conditions	Results	Remarks
Wimalasiri and Zou (2013)	SW-CNT/graphene nanosheets for CDI electrodes	25 ml/min feed flow, 780 mg/L NaCl aqueous solution, 25 °C, 2 V voltage	98% salt removal efficiency	-
Baek et al. (2014)	VA CNT + Epoxy	2–10 bar, 10–30 °C, with 600 rpm stirring speed, and 200 mg/L polyethylene oxide (PEO)	1100 ± 130 L/h.m^2^.bar. water flux and the PEO rejection of the VACNT membrane as examined was 78%	Membrane size Avg pore diameter (nm) 4.8 ± 0.9 Pore density(#/cm^2^) 6.8*10^10^
Dumée et al. (2010)	MD with 50 mg/m^2^ MW-CNT Matrix	Deionized water on cold side of MD, 35 g/L NaCl solution on hot side	99% salt rejection and 12 kg/${\mathrm{m}}^{2}$.h water flux rate	Water flux rate about 40 kg/m^2^.h at 28 kPa (Hot: 70 °C; Cold: 5 °C)
Li et al. (2014)	CNT/PES	60 Psi	VA-CNTs water transportation was 3 times the CNT/PES membrane and 10 times higher than the pure PES membrane under the same pressure	-
Shawky et al. (2011)	Nanocomposite of MW-CNT/polyamide (15 mg MW-CNT/g PA)	NaCl Aqueous solution (4000 ppm) at room temperature, 3.9 MPa	76 ± 1.1% salt rejection, 28 ± 0.8 L/h.m^2^.bar. Flux	-
Lee et al. (2015)	CNT	pressure at 1 bar	The water permeability through CNT millimeter thick UF membrane was 30,000 L/m^2^.h.bar	-
Bhadra et al. (2013)	Carboxylated CNTs (MWCNT-COOH/PVDF)	24 mL/min feed flow rate, 3.4 wt.% NaCl solutions, 1 L/min dry air, 60–90 °C	19.2 kg/h.m^2^ Productivity 99% salt rejection	The flow remained constant as the salt concentration increased, reaching 19.2 kg/h m^2^
Yan et al. (2012)	(0.4 SW-CNT/PANI)	NaCl solutions with an initial conductivity of 100 μS/cm, 20 ml/min feed flow, 1.2 V	The salt removal efficiency is 78.4% Regeneration rate of 100% for 0.4 SWCNT/PANI	
Wang et al. (2014)	CNT/Polypryrrole Electrodes	100 μS/mm NaCl aqueous solution	Saturated adsorption capacity was 96% and 43.99 mg/g for reactivation rate	-
Amini et al. (2013)	0.1% MW – polyamide/CNT	10 mM feed NaCl aqueous solution, 2 M NaCl draw solution, 23 °C, 240 kPa	96.7 L/h m^2^ water flux nearly 160% higher than TFC membrane	-
Park et al. (2014)	VA-CNT		The pure water permeabilities for the VA-CNT membrane 1000 ± 100 L/m^2.^h.bar	-
Zhao et al. (2014)	0.1% MW-polyamide/CNT	NaCl Aqueous solution (2000 ppm, 7 pH, 25 °C), 16 bars	28.05 L/h.m^2^ water flux and 90% salt rejection	With increase in the amount of MWNTs in the membrane, the water flux increased from 14.86 to 28.05 L/m^2^.h while the salt rejection decreased slightly
Jia et al. (2010)	CNT Forward osmosis	0.58 M NaCl aqueous solution (feed), 1 atm, and 300 K	100%-salt rejection for different sized CNTs	Water flux possessed by the CNT membranes tends to increase at first and then decrease with the increase of the CNT diameter in the investigating range

### *Nano-zeolite in **membrane distillation*

Nanosized zeolites are crystalline microporous solids with physicochemical characteristics like those of micron-sized crystals (Mintova et al. 2016). Nano-zeolite is characterized by a large surface area and easy shaping. Using nano-zeolite in desalination showed a promising future in increasing the productivity of membrane distillation. Anis et al. (2019) found that with 0.5% weight of nano-zeolite, the productivity increased by 34.2% with the salt rejection of 99.52%. Recently reported studies examining the effect of Nano-zeolite on the performance of membrane distillation systems are summarized in Table 4.

**Table 4:** Recent studies on the performance of membrane distillation systems using nano-zeolite

Reference	Material used	Operating conditions	Results
Anis et al. (2019)	Nano-zeolite	25 bars, 25,000 mg/L NaCl solution	For 0.5 wt.% nano zeolite: A salt rejection of 99.52% with a flux increase of 34.2%
Liu and Chen (2013)	Nano-zeolite	Aqueous NaCl (with initial NaCl concentration of 1 mol/L	100% rejection of salt, the permeability is about 2 × ${10}^{-9}$ m/Pa.s
Kim et al. (2013)	Nano-zeolite	-	98.8% Salt rejection and 37.8 L/ m^2^.h water flux

## Solar stills

### Overview

The use of solar distillers is one of the appropriate ways to address the problem of freshwater scarcity, but the main problem of solar distillers is the low rate of daily water production. The intensity of solar radiation has a direct impact on the productivity of solar distillers since the hot and dry climatic conditions characterize most of the remote regions that suffer from water scarcity and have a high solar intensity. The use of solar distillers can help these countries save drinking water. The performance of solar distillers is mostly affected by the rates of water evaporation and condensation on the glass surface, where the increase in the temperature difference between the basin water and the glass cover (condensing surface) helps to increase the water productivity of the solar distillers.

### Theoretical analysis of solar stills

Thermal analysis of the solar distillers was found to be dependent on the co-efficient of internal heat transfer and efficiency. The formula of these parameters was discussed below (Baskaran and Saravanane 2021):

The thermal efficiency η_th_ of the solar distiller depends on the entirety of hourly productivity ${\dot{m}}_{dw}$, latent heat of vaporization h_fg_, solar intensity I(t), and the projected area A of solar distillers which calculated as follows:

9 $${\eta }_{th}=\frac{\sum {\dot{m}}_{dw} {h}_{fg}}{\sum I\left(t\right)A}$$

Latent heat of vaporization h_fg_ and entirety of hourly productivity ${\dot{m}}_{dw}$ calculated by the following Eqs. (10), (11):

10 $${\mathrm{h}}_{fg}={10}^{3}\times [2501.897-2.407 {\mathrm{T}}_{\mathrm{w}}+1.192\times {10}^{-3} {\mathrm{T}}_{\mathrm{w}}^{2}-1.596\times {10}^{-5} {\mathrm{T}}_{\mathrm{w}}^{3}$$

11 $${\dot{\mathrm{m}}}_{\mathrm{dw}}=3600 \left(\frac{{\mathrm{h}}_{\mathrm{ew}}}{{\mathrm{h}}_{\mathrm{fg}}}\right)\mathrm{A }\left({\mathrm{T}}_{\mathrm{w}}- {\mathrm{T}}_{\mathrm{gi}}\right)$$

The co-efficient of evaporative heat transfer h_ew_ calculated by:

12 $${\mathrm{h}}_{\mathrm{ew}}=16.273 {\times {10}^{-3}\mathrm{ h}}_{\mathrm{cw}}\left[\frac{\left({P}_{w}- {P}_{g}\right)}{\left({\mathrm{T}}_{\mathrm{w}}- {\mathrm{T}}_{\mathrm{g}}\right)}\right]$$

The co-efficient of convective heat transfer h_cw_ calculated by:

13 $${\mathrm{h}}_{\mathrm{cw}}=0.884 {\left[\left({\mathrm{T}}_{\mathrm{w}}- {\mathrm{T}}_{\mathrm{g}}\right)+\frac{\left({P}_{w}- {P}_{g}\right)\left({\mathrm{T}}_{\mathrm{w}}+ 273\right)}{\left(268.9\times {10}^{3}\right)- {\mathrm{P}}_{\mathrm{w}}}\right]}^{1/3}$$

The co-efficient of radiative heat transfer h_rw_ calculated by:

14 $${\mathrm{h}}_{\mathrm{rw}}=\left(\frac{1}{{\varepsilon }_{w}}+\frac{1}{{\varepsilon }_{g}}-1\right)\upsigma \left[{\left({\mathrm{T}}_{\mathrm{w}}+273\right)}^{2}+{\left({\mathrm{T}}_{\mathrm{g}}+273\right)}^{2}\right]$$

where, ${\upvarepsilon }_{\mathrm{w}}={\upvarepsilon }_{\mathrm{g}}=0.9$, ${T}_{w}$ is basin water temperature, ${\mathrm{T}}_{\mathrm{g}}$ is glass cover temperature, and ${\mathrm{P}}_{\mathrm{w}}$ and ${\mathrm{P}}_{\mathrm{g}}$ are partial vapor pressures at free water surface and glass cover, respectively.

### Solar still performance improvement

Solar stills use the heat from the sun’s irradiance to evaporate the water and then condense it on a glass sheet to produce pure water. The main goal to improve the distillers performance is to increase the amount of heat absorbed by the solar still, this can be achieved by using thermal storage materials with high thermal conductivity to increase the absorbed heat and even store it; also preheating the feed flow to the solar still can improve the performance as it makes the process faster and requiring less sun irradiance; another way is to use the nanofluids due to high thermal conductivity of these materials.

### Preheating the feed flow of solar still

**Fig. 5:**
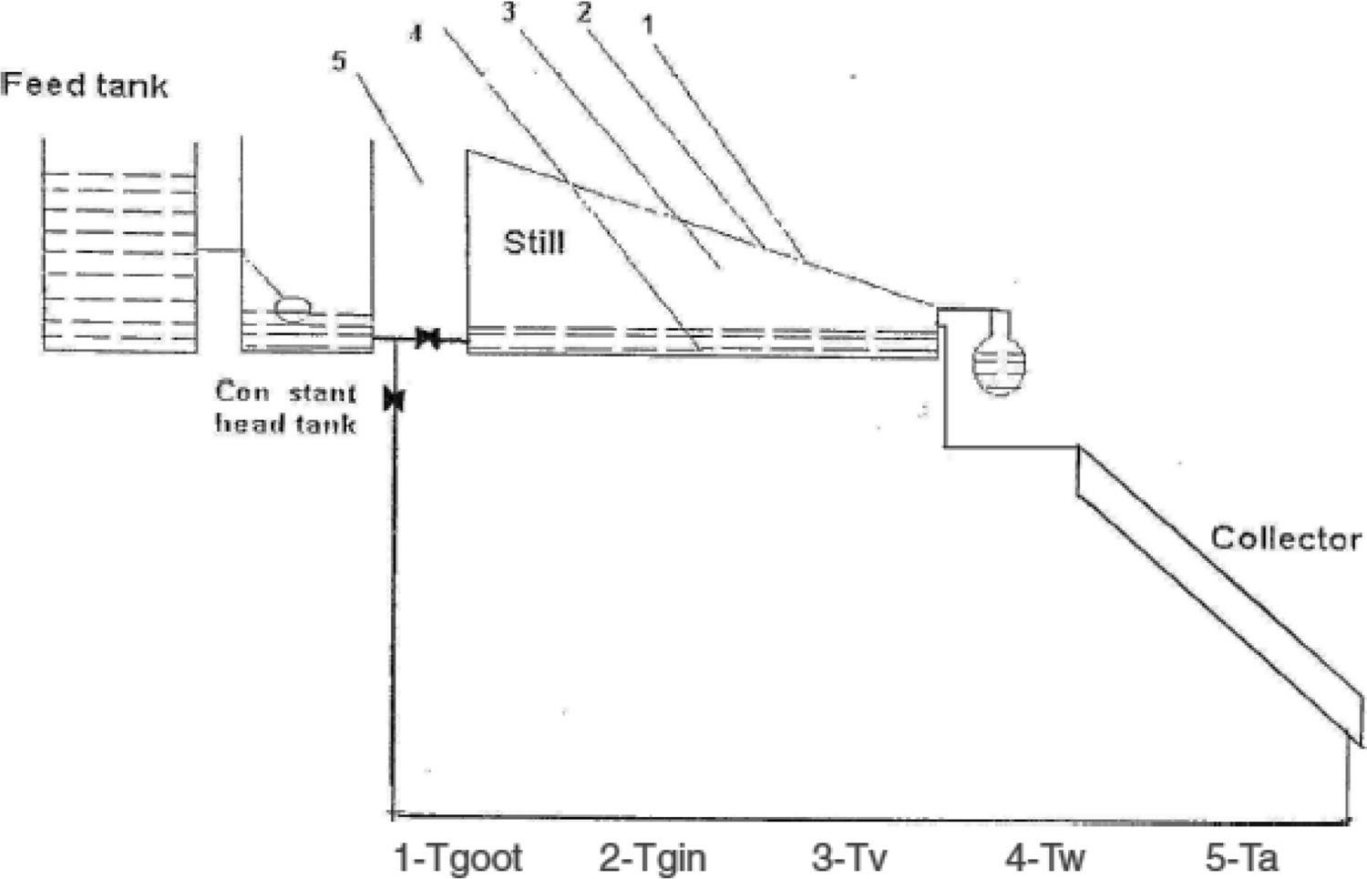
A solar still coupled with solar collector (Badran and Al-Tahaineh 2005)

**Fig. 6:**
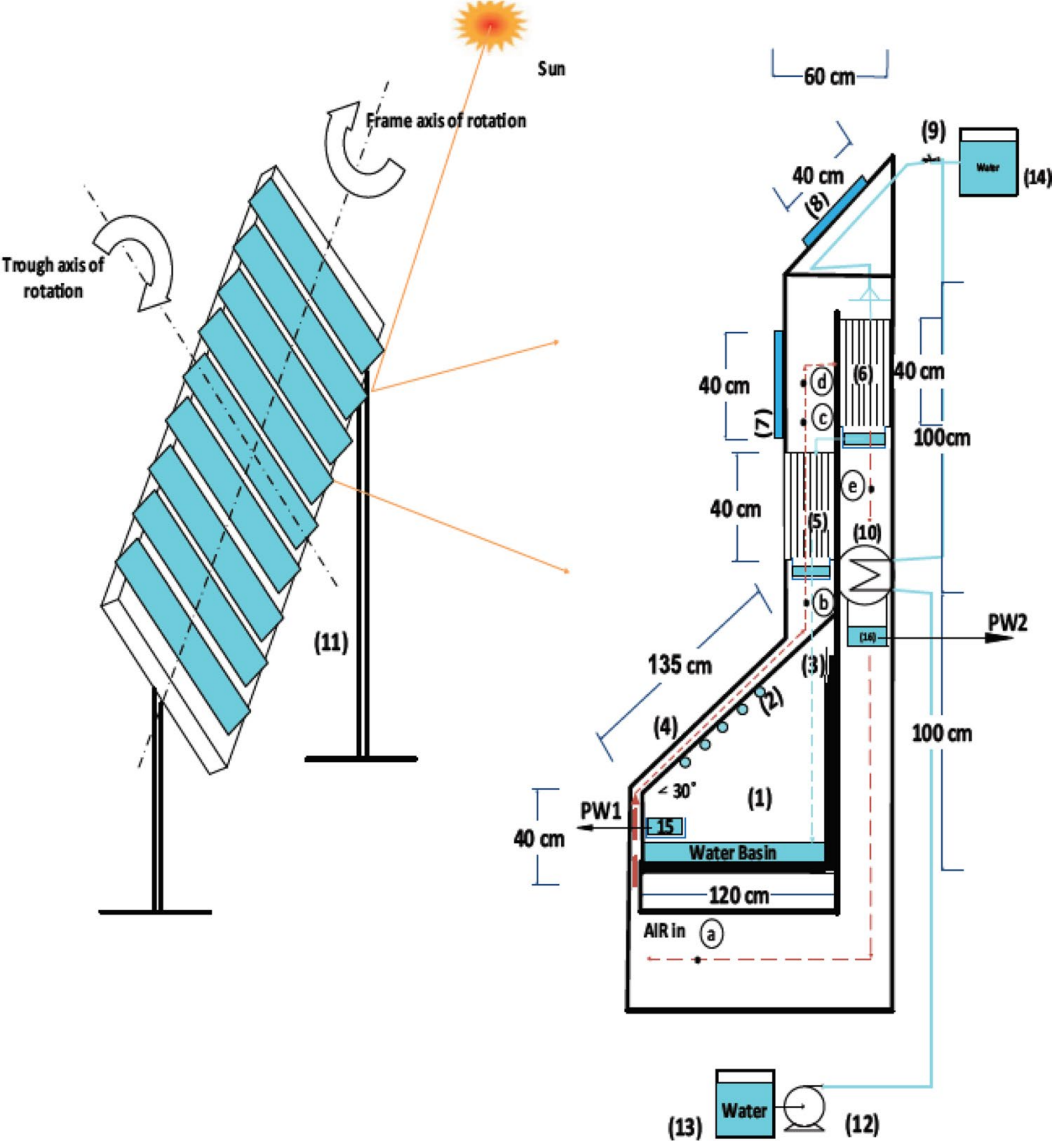
Layout of hybrid humidification-dehumidification/solar still integrated with photovoltaic panels and solar concentrators (Mahmoud et al. 2018)

Badran and Al-Tahaineh (2005) investigated the effect of using solar heaters on the production of the solar stills, Fig. 5; they found that coupling of a solar collector with distiller improved the production by 36%, as the productivity with solar collector 3510 mL and without the solar heater was 2240 mL. Mahmoud et al. (2018) studied the effect of adding a solar concentrator to increase the heat directed to the solar still integrated with humidification-dehumidification desalination system shown in Fig. 6; results showed that with a concentration ratio of 4 and a Basin water height of 0.01 m, the system yield was about 16.3 kg/${\mathrm{m}}^{2}$. Recently reported studies examining the effect of feed water preheating technologies on the performance of solar distillers are summarized in Table 5.

**Table 5:** Recent studies on the performance of solar distillers using feed water preheating technologies

Ref	Nature	Solar still type	Improvement-techniques	Results	Remarks
Mahmoud et al. (2018)	Experimental + Theoretical	Single slope	HDH desalination unit + solar photovoltaic/thermal (PV/T) panels combined with solar concentrator	• The system yield was about 17 kg/m^2^ at basin water temperature 80 °C • Electrical efficiency of PV/T reached 7.8% and 9.4% for the preheated water and air, respectively	The yield decreases with increases in the circulating air flow rate and the basin height
Sathyamurthy et al. (2015)	Experimental + Theoretical	Semi-circular	Semi-circular trough with baffles	The daily yield is 3 kg/m^2^ with daily efficiency of 38%	The daily yield improved by 16.66%
Kabeel and Abdelgaied (2019)	Experimental	Single slope	Photovoltaic/thermal panels with reflectors + air injection system	• The daily yield reached 6034 ml/m^2^ with an improvement of 40.98% • Overall efficiency reached 33.55% • Distillate water cost reached 0.014 $/L	Using an air injection system reduced the distillate water cost by 28.57% compared to reference distillers
Badran and Al-Tahaineh (2005)	Experimental	Single slope	Mirrors + Flat plate collector	The productivity increased by 36%	-
El-Sebaii et al. (2011)	Experimental + Theoretical	Single slope	Shallow solar pond as preheating unit	The maximum daily production reached 6.68 kg/m^2^ and average efficiency reached 47.54%	-
Fallahzadeh et al. (2020)	Experimental + Theoretical	Pyramid-shaped	Evacuated tube solar water collector	The daily yield reached 6970 ml/m^2^ and water cost reached 0.0137 $/L	-
Amiri et al. (2021)	Experimental + Theoretical	Single slope	Parabolic trough collector with tracking systems	Daily yield reached 8 L/m^2^ Energy efficiency reached 63%	-
Hassan et al. (2020)	Experimental	Single slope	Parabolic trough solar collector integrated with oil heat exchanger	The daily yield reached 8.77 L/m^2^; Thermal efficiency 40.3%; Water cost reached 0.01937 $/L	Use of these desalination systems mitigated CO_2_ compared to other desalination systems depending on fossil fuel
Alwan et al. (2020)	Experimental	Single slope	Flat plate solar water collector + rotating hollow drum	Productivity reached 5.5 L/m^2^ day with an improvement of 292%, and the distillate water cost reached 0.048 $/L	The yield improved by 292% compared to reference distillers
Azari et al. (2021)	Experimental	Single slope	V-groove solar air collector as preheating unit	The yield, energy, and exergy efficiencies improved by 170%, 170%, and 257%, respectively compared to reference unit; Water cost reached 0.03$/L	-
Sharma et al. (2022)	Experimental + Theoretical	Single slope	Evacuated tubular solar collector	Daily fresh water yield reached 7.1 kg/m^2^	
Thakur et al. (2021)	Experimental	Single slope	V-type solar concentrator	Daily yield reached 5.47 L/m^2^, energy efficiency reached 57.4%, and exergy efficiency reached 3.8%; water cost reached 0.0102 $/L	Use of V-shape concentrator as preheating unit and minimum water depth are useful to augment the highest performance

### Solar still with thermal storage mediums

**Fig. 7:**
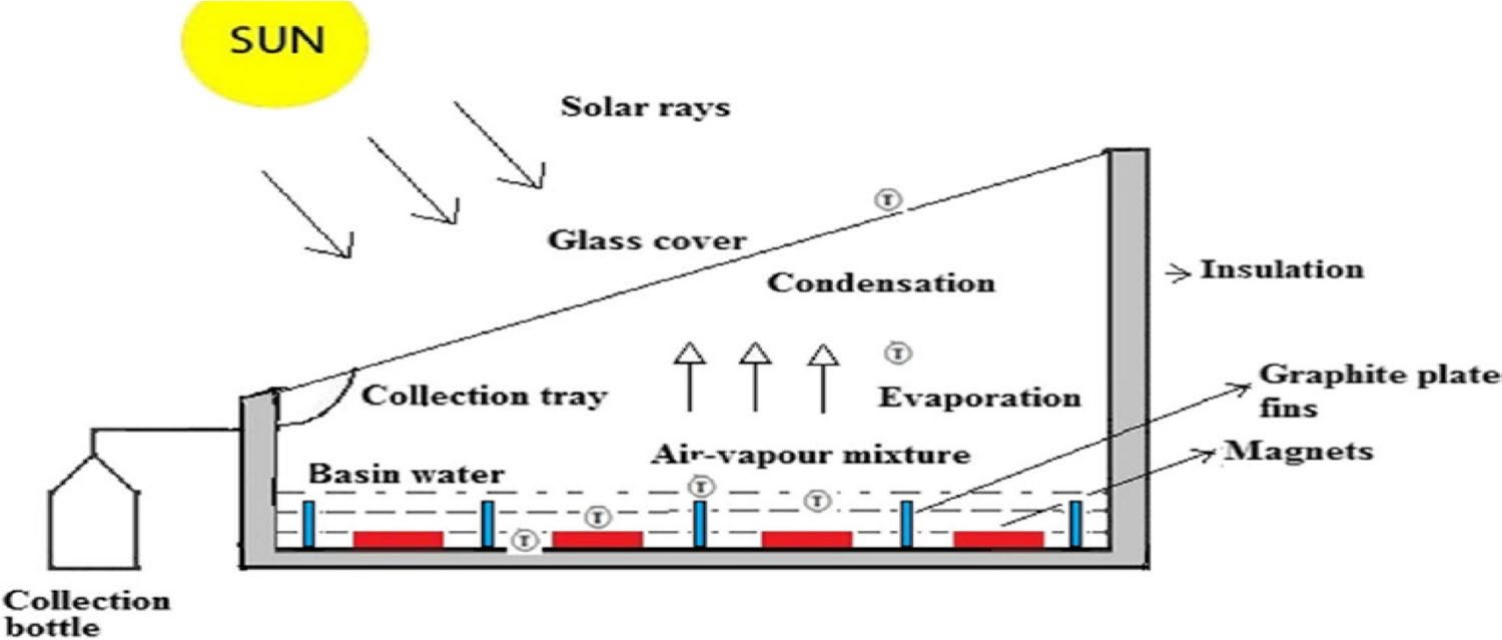
Single slope still with magnets and graphite plate fins (Dhivagar and Mohanraj 2021)

Thermal storage mediums are integrated with solar stills to increase the heat absorbed by the basin of the still and to work as a heat source at night. Dhivagar and Mohanraj (2021) used 16 magnets and 20 graphite plate fins shown in Fig. 7. The results conducted that the yield, energy, and exergy efficiency of graphite plate fins and magnet solar still (GPF-MSS) were increased by 19.6, 21.4, and 18.1%, respectively, compared with conventional solar still. Recently reported studies examining the effect of thermal storage mediums on the performance of solar distillers are summarized in Table 6.

**Table 6:** Recent studies on the performance of solar distillers using thermal storage mediums

Ref	Nature	Solar still type	Improvement-techniques	Results
Dhivagar and Mohanraj (2021)	Experimental + Theoretical	Single slope	Graphite plate fins as sensible storage materials and magnets	The average productivity of modified distiller was 23.8% higher than the reference unit The energy efficiency improved by 21.3% compared to reference unit
Kabeel et al. (2020a)	Experimental	Pyramid-shaped	Hollow circular fins and paraffin wax as energy storage materials (Solid/liquid heat capacity 2.95/2.51 kJ/kg °C)	The hollow fins utilization increases the cumulative yield to 5.75 L/m^2^ with 43% improvement PCM addition increases the daily yield to 8.1 L/m^2^ with 101.5% improvement
Kabeel and Abdelgaied (2017a)	Experimental	Single slope	Cylindrical parabolic concentrator + Paraffin wax as energy storage materials (Solid/liquid heat capacity 2.95/2.51 kJ/kg °C)	Freshwater daily productivity reached 10.77 L/m^2^ Distillate cost reached 0.1378 LE/L Daily efficiency reached 46%
Kabeel et al. (2021)	Experimental + Theoretical	Tubular	V-corrugated wick materials	V-corrugated wick materials improve the yield to 6010 ml/m^2^, with an improvement of 44.82% The energy efficiency reached 51.4%
El-Bialy (2014)	Experimental	Single slope	Floating absorber	The daily productivity improvement ratio was 42.2%, 15.2%, 20.1% and 17.2% when mica, stainless steel, aluminum and copper are used as floating absorbers
Sellamia et al. (2017)	Theoretical	Single slope	Heat storage blackened layers of sponge	A 0.5 cm sponge thickness improved a yield by 57.77%
Kabeel and Abdelgaied (2017b)	Experimental	Single slope	Coaxial pipes in basin	The daily yield improved by 97.8% The energy efficiency improved by 90.8%
Al-Harahsheh et al. (2022)	Experimental	Single slope	Solar collector as preheating unit + PV panel + Basin tube filled with sodium acetate trihydrate as storage materials (Solid/liquid heat capacity 2.79/3 kJ/kg °C)	The daily yield reached 9.7 L/m^2^ and the thermal efficiency reached 54.1% The overall improvement in distillate yield reached 400% compared to reference distiller
Maridurai et al. (2021)	Experimental	Single slope	Flat plate solar collector + Paraffin wax as energy storage materials (heat capacity 2.2 kJ/kg °C)	The daily yield improved by 22% compared to reference unit
Shehata et al. (2020)	Experimental	Single slope	Ultrasonic waves, reflectors, paraffin wax as energy storage materials (Solid/liquid heat capacity 2.95/2.51 kJ/kg °C), and evacuated solar collector	Daily yield reached 7.4 L/m^2^; Distillate cost reached 0.037$/L; Thermal efficiency reached 49%; Distillate yield improved by 44% compared to reference distillers
Malik et al. (2021)	Theoretical	Weir-type	Paraffin wax as energy storage materials (solid/liquid heat capacity 2.95/2.51 kJ/kg °C)	Exergy efficiency and annual distilled water improved by 1.47% and 4.35% compared to reference unit
Abu-Arabi et al. (2020)	Theoretical	Single slope	Flat plate solar collector + sodium acetate trihydrate as energy storage materials (solid/liquid heat capacity 2.79/3 kJ/kg °C) + glass cover cooling	Daily yield reached 7.4 L/m^2^ and thermal efficiency reached 49.2%
Abdelgaied et al. (2022)	Experimental	Hemispherical	Paraffin wax as energy storage materials (solid/liquid heat capacity 2.17/3.06 kJ/kg °C) and CuO-water–based nanofluid	Distillate productivity improved by 60.41%, and daily thermal efficiency reached 63.61% Water cost 0.0065 $/L

### Solar still with nanomaterials

#### ***Nano Al***_***2***_***O***_***3***_

Xia et al. (2016) conducted the influences of adding nano Al_2_O_3_ and Ti ${\mathrm{O}}_{2}$ on heat transfer rate; they found that that the thermal conductivity and dynamic viscosity of Al_2_O_3_ and Ti ${\mathrm{O}}_{2}$ nanofluids are both improved with the increase of particle volume fraction. Kabeel et al. (2014) experimented the addition of Al_2_O_3_ to a single-sloped solar still with external condenser to improve the condensation of the evaporated water of the still; results have shown a 116% improvement in the productivity of the still. Recently reported studies examining the effect of nano Al_2_O_3_ on the performance of solar distillers are summarized in Table 7.

**Table 7:** Recent studies on the performance of solar distillers using nano Al_2_O_3_

Ref	Nature	Solar still type	Material used	Results
Kabeel et al. (2014)	Experimental	Single slope	Al_2_O_3_	With the external condenser, the solar still water productivity increased by about 116% Water cost 0.041 $/L
Sahota and Tiwari (2016)	Theoretical	Double slope	0.12% Al_2_O_3_ concentration	The enhancement of yield has been found to be 12.2%
Chaichan and Kazem (2018)	Experimental	Single slope	Paraffin wax with a nano-Al_2_O_3_	Distillate yield improved by 60.53%
Modi et al. (2021)	Experimental	Single-basin dual-slope	0.1% Al_2_O_3_	Use of Al_2_O_3_ improved the yield by 28.53%
Kabeel et al. (2017a)	Numerical	Single slope	0.2% Al_2_O_3_ and 0.2% Cu_2_O	The daily efficiency improved by 73.85% and 84.16% for Al_2_O_3_ and Cu_2_O nanoparticles, respectively
Shanmugan et al. (2018)	Experimental + Theoretical	Single slope	Al_2_O_3_	The daily yield was 7.460 kg/m^2^ in summer and 4.120 kg/m^2^ in winter

#### Nano CuO

Kabeel et al. (2017a) tested experimentally the effect of CuO concentration on the distillate of the solar still; they found that with 10% CuO concentration, the distillate increased by 16%, and by increasing the CuO concentration to 40%, the distillate increased by 40%. Recently reported studies examining the effect of CuO nano material on the performance of solar distillers are summarized in Table 8.

**Table 8:** Recent studies on the performance of solar distillers using CuO nanomaterial

Ref	Nature	Solar still type	Material used	Results
Modi et al. (2021)	Experimental	Dual slope	0.1% CuO concentration	Use of CuO improved the yield by 58.25%
Kabeel et al. (2017b)	Experimental	Single slope	10 to 40% CuO concentration	Utilizing CuO nanoparticles boosted the distillate by 16% and 25% at CuO concentration of 10% and 40%, respectively
Gupta et al. (2016)	Experimental	Single slope	0.12% CuO concentration	Solar still with added nanoparticles produced 3445 ml/m^2^ per day at water depth of 5 cm
El-Gazar et al. (2021)	Experimental + Theoretical	Single slope	Al_2_O_3_ + CuO 0.025% for each	Enhancement in the still output yield reached 27.2% in summer and 21.7% in winter compared to reference still
Attia et al. (2021)	Experimental		CuO-water–based nanofluid	Daily yield reached 6.8 L/m^2^ with an improvement of 76.6% Water cost 0.0066 $/L

#### Other nanomaterials

Cuprous oxide (Cu_2_O), titanium dioxide (TiO_2_), potassium permanganate (KMnO_4_), zinc oxide (ZnO), Fe_2_O_3_ and SnO_2_ nanomaterials can also be used to enhance the distiller yield. Elango et al. (2015) compared between using of different nanomaterials on the performance of a single slope solar still, Al_2_O_3_, ZnO, Fe_2_O_3_ and SnO_2_ were tested, results showed that the yield improved 29.95, 12.67, and 18.63% for Al_2_O_3_, ZnO, and SnO_2_ nanofluids, respectively. Recently reported studies examining the effect of other nanomaterials on the performance of solar distillers are summarized in Table 9.

**Table 9:** Recent studies on the performance of solar distillers using other nanomaterials

Ref	Nature	Still type	Material used	Results
Omara et al. (2015)	Experimental	Single slope	Al_2_O_3_ + cuprous oxide	The yield enhanced by 285.10% and 254.88% for using cuprous and Al_2_O_3_ nanoparticles, respectively
Kabeel et al. (2019a, b)	Numerical	Single slope	0.02% cuprous oxide	The daily yield improved by 106.86%
Shanmugan et al. (2020)	Experimental + Theoretical	Single slope	TiO_2_	In summer: average daily productivity was 7.89 L, and average efficiency of a system was 36.69% In winter: average daily productivity was 5.39 L, and average efficiency of a system was 57.16%
Kabeel et al. (2019a)	Experimental	Pyramid solar still	TiO_2_ black paint coated solar still	The distilled yield improved by 6.1%
Nijmeh et al. (2005)	Experimental	Dual slope	KMnO_4_	The improvement in energy efficiency reached 26%
Elango et al. (2015)	Experimental	Single slope	Al_2_O_3_, ZnO, Fe_2_O_3_ and SnO_2_	The yield improved 29.95, 12.67, and 18.63% for Al_2_O_3_, ZnO, and SnO_2_ nanofluids, respectively
Kabeel et al. (2018)	Experimental	Single slope	Graphite nanoparticles	The daily yield reached 7.73 L/m^2^
Kabeel et al. (2019b)	Experimental	Single slope	Paraffin wax and graphite nanoparticles	The daily yield reached to 7.123, 7.475, 7.937, 8.249, and 8.52 L/m^2^ for 0.0, 5, 10, 15, and 20% graphite mass concentrations, respectively
Kabeel et al. (2020b)	**-**	Stepped solar still	Graphite and PCM + internal reflectors and evacuated tube collector	The daily distillate productivity varied between 13.6 and 13.62 L/m^2^
Rufuss et al. (2018)	Experimental	Single slope	PCM + TiO_2_, CuO, and GO nanoparticles	Add TiO_2_, CuO, and GO nanoparticles to PCM improved the yield to 3.92, 4.94, 5.28 and 3.66 L/$${\mathrm{m}}^{2}$$ per day, respectively Water cost reached 0.026 $/L
Nazari et al. (2019)	Experimental + Theoretical	Single slope	Cu_2_O nanofluid + thermoelectric cooling channel	Use of 0.08% Cu_2_O improved the yield and energy efficiency by 82.4% and 81.5%, respectively Water cost reached 0.021 $/L
Kabeel et al. (2017c)	Numerical	Single slope	Cu_2_O + Al_2_O_3_	Use of 0.02% Cu_2_O and Al_2_O_3_ improved the daily yield to 4090 ml/m^2^ and 2875 ml/m^2^, respectively
Arani et al. (2021)	Experimental		SiO_2_ nanoparticles	Productivity improved by 55.18%, and water cost reached 0.012 $/L

## Conclusion

In addition to the importance of both membrane desalination plants and solar stills in the field of water desalination, this paper aims to provide a comprehensive review of the most important recent studies aimed at improving performance that was conducted on both membrane desalination plants and solar stills. The improvement axes that were carried out on both the membrane desalination plants and the solar stills were categorized according to three axes that are very effective and have a direct impact on the performance of the membrane desalination plants and the solar stills, which are as follows: feed water preheating technologies, thermal storage materials, and nanoparticles. This survey focuses on showing the impact of the previous improvement axes on pure water productivity, energy efficiency, and the cost of producing pure water. The most important results can be outlined as follows:

• The contribution of solar heat and waste heat used in the operation of the process leads to a lower cost of water production as well as making the desalination system more competitive, sustainable, and economically viable for small and remote applications.
• Permeate pure water through the membrane was increased by 52 g/m^2^/min for increasing the feed water temperature from 30 to 60 °C.
• Using waste heat and solar thermal energy reduced the cost of pure water produced from the membrane distillation plant from 6.80 $/m^3^ to 1.6 $/m^3^ compared to a membrane distillation plant that operated with standalone grid electricity.
• The overall system efficiency of the membrane distillation plant improved from 46.6% to 61.8% for utilizing the pre-cooling unit on the permeate flow loop before entering the membrane unit.
• The pure water productivity of the membrane distillation system will be improved by a rate varying between 33.11 and 43.18% compared to cases without PCM.
• Use of thermal storage materials improved the cumulative yield and the gain output ratio of membrane distillation units by a rate up to 43.2% and 34.4%, respectively.
• With an increase in the amount of MWNTs in the membrane, the water flux increased from 14.86 to 28.05 L/m^2^.h, while the salt rejection decreased slightly.
• For utilizing 0.5 wt.% nano-zeolite, the salt rejection reached 99.52% and the water flux increased by 34.2%.
• Using the solar collector as a feed water preheating unit is the effective choice that increases the pure water productivity and energy efficiency with rates reaching 40.98% and 57.4%, respectively, and reduces the cost of producing pure water to a rate reached 0.0102 $/L.
• The utilization of nanofluid improved the cumulative productivity of solar stills by a rate up to 116%, and also the use of thermal storage materials (PCM) improved the cumulative productivity of solar stills at a rate of 105.5%.

## Recommendation and future scope

In the processes of reducing the electricity consumption rates in membrane distillation units, various modifications were shown in many of the recent studies that were conducted, all of which aim to reduce the electrical power consumption rates, but there is still room for brainstorming. Also in the processes of enhancing the cumulative yield and raising the efficiency of solar stills, various modifications were presented in many of the researches that were conducted, all of which aim to enhance the cumulative yield and raise the efficiency of solar stills, but there is still room for a brainstorm. The following are the most important recommendations that could be useful for further study, correction, and modification of membrane distillation units and solar stills.

• The heat released from the waste outlets in industries and exhaust of the engine is the most effective choice as a feed water preheating unit to improve the performance of membrane distillation units.
• Development of the new combination of the membrane distillation and solar still to achieve the highest performance and lower cost.
• Incorporating high-performance nanomaterials, thermal storage materials, and feed water preheating units in the water treatment process are good choices to achieve the highest performance and lower cost.
• Future studies are also required to be focusing more on the effects of novel combinations of effective modifications in order to determine the best designs of the solar stills that achieve the highest performance and lower cost.

## Data Availability

Not applicable.

## Nomenclature

ASBS: Active single basin solar still
CDI: Capacitive deionization
CNT: Carbon nanotube
FO: Forward osmosis
GOR: Gain output ratio
GPF: Graphite plate fins
MSS: Magnet solar still
MW-CNT: Multi-wall Carbon nanotube
PCM: Phase change material
PV: Photovoltaic
SSA: Spectrally selective absorber
SSP: Shallow solar pond
SW-CNT: Single wall carbon nanotube
TDCMD: Tubular direct contact membrane desalination
VACNT: Vertically aligned carbon nanotube
